# Clinical management of patients with genetic obesity during COVID-19 pandemic: position paper of the ESE Growth & Genetic Obesity COVID-19 Study Group and Rare Endo-ERN main thematic group on Growth and Obesity

**DOI:** 10.1007/s12020-021-02619-y

**Published:** 2021-01-29

**Authors:** Cornelis Jan De Groot, Christine Poitou Bernert, Muriel Coupaye, Karine Clement, Stavroula A. Paschou, Evangelia Charmandari, Christina Kanaka-Gantenbein, Martin Wabitsch, Emilie P. Buddingh, Barbara Nieuwenhuijsen, Ljiljana Marina, Gudmundur Johannsson, E. L. T. Van Den Akker

**Affiliations:** 1 Pediatric Endocrinology and Obesity Center CGG Erasmus MC, Rotterdam and Willem Alexander Children’s Hospital, LUMC, Leiden, the Netherlands; 2 Assistance Publique-Hôpitaux de Paris, Reference Center for Rare Diseases (PRADORT, Prader-Willi Syndrome and other rare obesities), Nutrition Department, Pitié-Salpêtrière hospital, Paris, France; 3 Sorbonne Université/INSERM, Nutrition and obesities; systemic approaches (NutriOmics) research Unit, Paris, France; 4 Assistance Publique-Hôpitaux de Paris, Reference Center for Rare Diseases (PRADORT, Prader-Willi Syndrome and other rare obesities), Nutrition Department, Pitié-Salpêtrière and Louis-Mourier hospitals, Paris, France; 5 Assistance Publique-Hopitaux de Paris, Nutrition Department, Pitie-Salpetriere Hospital, Paris, France; 6 Division of Endocrinology, Diabetes and Metabolism, Department of Clinical Therapeutics, Alexandra Hospital, School of Medicine, National and Kapodistrian University of Athens, Athens, Greece; 7 Pediatric and Adolescent Endocrinology, National and Kapodistrian University of Athens Medical School, “Aghia Sophia” Children’s Hospital, Athens, Greece; 8 Division of Endocrinology, Center for Clinical, Experimental Surgery and Translational Research, Biomedical Research Foundation of the Academy of Athens, Athens, 11527 Greece; 9 Division of Endocrinology, Diabetes and Metabolism, First Department of Pediatrics, Medical School, National and Kapodistrian University of Athens, Aghia Sophia Children’s Hospital, Athens, Greece; 10 Division of Pediatric Endocrinology and Diabetes Department of Pediatrics and Adolescent Medicine, University Medical Center Ulm, Ulm, Germany; 11 Willem-Alexander Children’s Hospital, LUMC, Leiden, the Netherlands; 12 Faculty of medicine, UMC Utrecht, Utrecht, the Netherlands; 13 Assistant Professor Clinic for Endocrinology, Diabetes and Metabolic Diseases, Clinical Centre of Serbia, Faculty of Medicine, University of Belgrade, Belgrade, Serbia; 14 Institute of Medicine, Sahlgrenska Academy, University of Gothenburg and Department of Endocrinology, Sahlgrenska University Hospital, SE-413 45, Gothenburg, Sweden; 15 Pediatric Endocrinology and Obesity Center CGG, Erasmus MC Sophia Children’s Hospital, Rotterdam, the Netherlands

**Keywords:** Monogenic obesity, Genetic obesity, Obesity syndrome, COVID-19, SARS-CoV-2

## Abstract

This article aims to provide guidance on prevention and treatment of COVID-19 in patients with genetic obesity. Key principals of the management of patients with genetic obesity during COVID-19 pandemic for patients that have contracted COVID-19 are to be aware of: possible adrenal insufficiency (e.g., POMC deficiency, PWS); a more severe course in patients with concomitant immunodeficiency (e.g., LEP and LEPR deficiency), although defective leptin signalling could also be protective against the pro-inflammatory phenotype of COVID-19; disease severity being masked by insufficient awareness of symptoms in syndromic obesity patients with intellectual deficit (in particular PWS); to adjust medication dose to increased body size, preferably use dosing in m2; the high risk of malnutrition in patients with Sars-Cov2 infection, even in case of obesity. Key principals of the obesity management during the pandemic are to strive for optimal obesity management and a healthy lifestyle within the possibilities of the regulations to prevent weight (re)gain and to address anxiety within consultations, since prevalence of anxiety for COVID-19 is underestimated.

## Introduction

In the first half of 2020 there has been a rapid increase of patients diagnosed with a new disease, Coronavirus Disease 2019 (COVID-19), caused by the Severe Acute Respiratory Syndrome Coronavirus 2 (SARS-CoV-2). On March 11, 2020, the World Health Organization declared COVID-19 a public health emergency of international concern. On 22-10-2020, 40,665,438 confirmed cases and 1,121,843 confirmed deaths were reported [1] as a result of this new disease. COVID-19 is primarily a respiratory disease with an often mild presentation with symptoms of a common cold, but more specific symptoms related to the infection including loss of smell and/or taste and shortness of breath may also occur [2]. A significant number of patients, however, develops severe respiratory complaints with acute respiratory distress syndrome, often complicated by bacterial and fungal superinfections, with a high mortality rate [2]. Furthermore, the disease can also disseminate into a multi-system disease with myocarditis, liver and kidney failure, widespread thromboembolic complications as hallmark features, which can lead to multiple organ failure and subsequent mortality [2]. Multiple risk factors associated with a severe disease course have been identified such as age over 65, male sex, obesity, type 2 diabetes, hypertension and an immunocompromised state [3–7].

The increased risk of a severe course of COVID-19 in obesity is particularly of great concern in subjects with severe obesity caused by monogenic disease or obesity syndromes, since the risk of a severe course of COVID-19 increases with increasing BMI [3]. To date, very little is known on the specific risks that adults and children with these diseases may have on a severe course of COVID-19 and whether there should be extra measures taken to protect or treat people suffering from this heterogeneous group of diseases. Therefore, this position paper aims to support clinicians and healthcare staff on the optimal management of patients with monogenic obesity and obesity syndromes under the circumstances of the pandemic due to COVID-19. Given the scarcity of research on COVID-19 and these specific diseases, the advice will be based on research of COVID-19 and obesity in general and the pathophysiological knowledge of these diseases combined with expert opinion based on the specific expertise of the Rare Endo-ERN ESE Growth & Genetic Obesity COVID-19 Study Group.

## Genetic obesity: definition, phenotype and treatment

In the last three decades, increasing possibilities of genetic testing have led to the identification of various genetic causes of severe obesity. Genetic obesity (Orpha code: 77828) is a collective term for both monogenic and syndromic obesity. Genetic obesity disorders are severe and disabling disorders that form a heterogeneous group of conditions. They are classically divided into non-syndromic (Orpha code: 98267) and syndromic obesity (Orpha code: 240371). Non-syndromic genetic obesity disorders are often caused by a single gene defect leading to Leptin (LEP) deficiency, leptin receptor (LEPR) deficiency, pro-opiomelanocortin (POMC) deficiency, melanocortin 4 receptor (MC4R) deficiency or proprotein convertase subtilisin/kexint type 1 (PCSK1) deficiency. Recently, other variants of genes involved in or regulating the melanocortin pathway and leading to early and severe obesity such as MC4R regulatory protein, melanocortin receptor accessory protein 2 (MRAP2), adenylate cyclase 3 (ADCY3), steroid coreceptor activator-1 (SRC-1) or kinase suppressor of ras 2 (KSR2) [8]. This leads to a defective LEP-melanocortin pathway in the hypothalamus, which is a key regulator of body weight, satiety and energy expenditure (14,15). Although early onset severe obesity (<5 years) and hyperphagia are the most common presenting features in these diseases, these patients can be affected by complex somatic conditions, including obesity comorbidities. In particular, several other endocrine disorders may also be present, such as hypothyroidism or hypogonadism in LEP and LEPR patients [9] or adrenal insufficiency in POMC and PCSK1 patients [10, 11]. LEP and LEPR deficiencies often lead to impaired immunity [12, 13]. These could be relevant in the light of COVID-19 and will be discussed in more detail later in this paper.

Syndromic genetic obesity differs from the non-syndromic diseases, as they have additional symptoms, apart from obesity [14–16], with intellectual disability, motor developmental delay and behavioural problems, such as autism being the most frequent features [14]. Both non-syndromic and syndromic obesity are more often diagnosed in children than in adult patient groups. Syndromic obesity entails a broad variety of syndromes with obesity as a hallmark. Prader–Willi Syndrome (PWS) is the most frequent and the most well-known syndrome within this group and is known to present with feeding difficulties and hypotonia in the first year of life with subsequent rapid weight gain caused by hyperphagia in infancy and behavioural problems like tantrums and autism. There are, however, many other syndromes such as Bardet–Biedl syndrome (BBS), Alström syndrome (ALS), pseudohypoparathyroidism and Cohen syndrome which present with infant or childhood onset obesity and concomitant symptoms such as developmental delay, specific visual or hearing impairments (e.g., ALS and BBS), endocrinopathies (e.g., ALS and BBS) and immunological problems (e.g., Cohen syndrome) [14, 15]. More recently, the Prader–Willi-like syndrome (among others associated with MYT1I, SIM1 and MAGEL mutations) and the 16p11.2 deletion syndrome have been described to be frequently accompanied by early onset obesity [17, 18].

Identifying genetic obesity requires a structured approach and diagnosis is crucial, not only for optimal treatment of the accompanying symptoms such as visual and hearing impairment and endocrinopathies, but also because the obesity accompanying these diseases requires a tailored and life-long approach [14]. For example, dietary expertise in the light of lowered resting energy expenditure or specific expertise on guidance of behavioural problems may be needed. Furthermore, for some of these diseases and syndromes, weight lowering drugs targeting hyperphagia such as setmelanotide are being introduced to the market that can overcome the defects in the LEP-melanocortin pathway and lead to significant weight loss [19, 20]. In a recent report liraglutide also showed promising results in patients with MC4R deficiency, and further research is ongoing evaluating these effects [20, 21].

This review will concentrate on those diseases and syndromes defined as non-syndromic and syndromic obesity by the orphanet classification system [22] and will not entail other forms of hypothalamic obesity, caused by structural lesions and/or their treatment, such as obesity after craniopharyngioma surgery or meningitis.

## The link between obesity and severe course of COVID-19

Since the beginning of the COVID-19 pandemic, several risk factors for a severe course of the disease have been identified. Besides age [7] and male sex [7], for example, diabetes and hypertension are important predictors of disease outcome [23]. Obesity also showed a strong association with severity of the course of COVID-19, independent of the comorbidities that are associated with obesity [7, 24]. Not only is obesity associated with an increased risk of hospital admission [3, 5, 7, 25], but also with the risk of intensive care admission [4, 25], the need for mechanical ventilation [7] and mortality [7, 25, 26]. The effect size of these relations is considerable. For example, a prospective study conducted in France on 5795 patients aged 18–79 years found that obesity doubled the mortality in patients hospitalized with COVID-19. The odd ratio was 1.9 for grade I obesity but was 2.8 and 2.6 for grade II and grade III, respectively [27].

Furthermore, data from patients with a COVID-19 infection (*n* = 265) treated at the intensive care unit at 6 University Hospitals in the USA have shown a significant inverse correlation between age and BMI. Indicating that younger patients, including some adolescents, had an increased risk of ICU admittance with increasing BMI when infected with COVID-19 [28].

The latter finding is of particular importance for paediatric patients with COVID-19, although in children, the risk of a severe course of obesity is generally low [29]. The limited available evidence, however, does show a possible link between obesity and disease severity, even in this age group [30] and the risk of a severe disease course increases with increased severity of obesity [7, 31], as was also shown in adolescent and young adult age categories [3, 5, 28]. The latter finding is of specific importance for monogenic and syndromic obesity since the majority suffers from severe obesity [14]. Furthermore it should be noted weight might play a role in the suggested link between SARS-CoV-2 and Kawasaki or Kawasaki like disease (recently named Paediatric Multi-system Inflammatory Syndrome temporally associated with COVID-19, PIMS-TS or Multi Inflammatory Syndrome in Children temporally related to COVID-19, MIS-C), with an unusual high incidence in the first months of 2020 and evidence of past or present infection with SARS-CoV-2 in most patients [32]. A recent meta-analysis showed that patients with this phenotype were overweight or obese in over 50% of the cases, again pointing towards an increased risk for overweight or obese children [33]. Whether this disease entity can also be linked to severity of obesity remains speculative. It is interesting that COVID-19 is not the first pandemic linking obesity to severity of disease. Although corona is a different virus than influenza, in the H1N1-influenza virus epidemic 10 years ago, with an CDC estimated 41-85 million infected people, severe obesity (defined as BMI > 40) was statistically associated with hospitalization among adults, independent of the presence of chronic medical conditions, and 33–55% of hospitalized patients with H1N1influenza infection were obese [34].

There are several possible pathways that could explain why obesity per se is associated with a more severe course of COVID-19 infection. These plausible, but speculative, pathways are discussed shortly.

1. Angiotensin converting enzyme (ACE). Obesity is associated with an imbalance in the renin-angiotensin-aldosterone system. This imbalance is characterized by an overexpression of angiotensin 2 (ANG2). ANG2 can be converted to angiotensin 1–7 (Ang1–7) by Angiotensin converting enzyme (ACE2) [35]. ANG2 has a pro-inflammatory effect, while ANG1–7 has an anti-inflammatory effect. It is therefore likely that this imbalance also contributes to the probability to develop a pro-inflammatory dysregulated immune response. SARS-CoV-2 enters the host cell by binding to ACE2-receptor. This happens mostly in the lungs; however, the ACE2-receptor is also expressed in the adipose tissue, which is abundant in individuals with obesity. This raises the question whether adipose tissue can be infected by SARS-CoV-2 and could therefore function as a reservoir for viral spread. This could result in an increased viral load and extended cytokine activation in the already low-grade inflamed adipose tissue [36].
2. Inflammatory dysregulation (often referred to as a cytokine storm) is an important driver of the more severe course of COVID-19 [2, 7, 37]. Obesity is a disease with chronic low-grade systemic inflammation with a disturbed balance between pro- (e.g., IL-6, TNF-alpha, IL-1beta) and anti-inflammatory (e.g., adiponectin) cytokines, leaning towards an overall pro-inflammatory state [37–39]. Infection with coronaviruses leads to an activation of the inflammasome and a subsequent increase in expression of IL1-beta [40]. Inflammasomes are cytosolic multiprotein oligomers of the innate immune system responsible for the activation of inflammatory responses. Since patients with obesity have an increased expression of the inflammasome component NLRP3 [41], infection with SARS-CoV-2 probably leads to an exaggerated pyroptotic response in patients with obesity, with features of macrophage activation syndrome. In addition, obese patients have defective adaptive immunity as evidenced by the increased numbers of T regulatory cells [42], which could result in a slower viral clearance and a prolonged activation of the innate immune system, thereby contributing to the immunopathology of severe COVID-19.
3. Obesity is linked to further metabolic and other severe impairments, such as type 2 diabetes, hypertension, thrombogenic risk, and lung impairment. These conditions all decrease the ability to cope with COVID-19 [43].
4. Viral shedding. The concept that patients with obesity might have increased viral shedding of SARS-CoV-2 comes from studies on the influenza A virus. It was found that obese patients are more contagious than lean patients. This is due to several factors. First, symptomatic obese patients shed influenza A virus 42% longer than lean patients, probably due to their altered immune response [44]. Second, BMI positively correlates with viral load in exhaled breath in patients with influenza. It is, however, not yet known whether the load of SARS-CoV2 in exhaled breath of obese COVID-19 patients is similarly increased as compared to lean patients [45].
5. Vaccine effectiveness. An effective vaccine for SARS-CoV-2 is highly anticipated. However, individuals with obesity are known to have a decreased influenza vaccine effectiveness, probably due to alterations in the immune system [46]. It is possible that this effect can also occur in obese individuals when vaccinated against SARS-CoV-2, leading to decreased prevention of COVID-19 in obese patients.
6. LEP. Obesity is characterized by higher LEP and lower adiponectin concentrations. LEP is a pro-inflammatory adipokine, while adiponectin is an anti-inflammatory adipokine [47]. In addition to this, LEP has an immune regulatory role. Intact LEP signalling is needed for a normal regulatory T-cell response, which has also been suggested to be one of the immunological linked to disturbances involved in the pathogenesis of the cytokine storm seen in patients with COVID-19 [19, 27].
7. Respiratory dysfunction. Obesity is associated with pulmonary dysfunction, with increased airway resistance, impaired gas exchange and low lung volume and muscle strength. Furthermore, abdominal obesity leads to decreased diaphragmatic excursion [48]. All these factors might contribute to the increased risk of a severe course COVID-19, but also with hypoventilation associated bacterial superinfections and difficulties applying mechanical ventilation in an ICU setting.
8. Bariatric surgery (BS). Little is known about the post BS condition and COVID-19 but this aspect was assessed in a cohort of 738 patients albeit with common obesity who underwent BS in France. The prevalence of likely COVID-19 and its risk factors was evaluated and revealed that in patients followed-up after BS, the disease occurred in 8.4% of the patients among whom 6.4% had a severe form requiring hospitalization (1.6% died). While the number of likely infection by SARS-CoV-2 was apparently similar to the general population in a similar geographic area, the COVID-19 likely group had a higher proportion of persistent type 2 diabetes at the last follow-up and showed a higher percentage of weight loss since the bariatric intervention. In this group, severe forms of COVID-19 requiring hospitalization were associated with persistent type 2 diabetes. This illustrates the particular attention that must be paid to patients who have lost weight but with the persistence of type 2 diabetes in this pandemic context [49].

## Are patients with genetic obesity at increased risk of a severe course in COVID-19?

In contrast to the extensive literature on obesity contributing to increased risk of a severe course of COVID-19 [37], literature on genetic obesity and COVID-19 is lacking. For PWS, according to a survey on COVID-19, performed by the IPWSO, 16 COVID-19 cases were reported, nevertheless, with mild symptomatic infections [50].

To identify possible risk factors of a more severe course of COVID-19 that are specific for patients with genetic obesity, there are a number of characteristics of these diseases that we need to consider.

- Severity of obesity. As mentioned above, this group of patients as a whole tends to have more severe obesity and the risk of a severe course of COVID-19 increases with increasing obesity class [3, 5, 7, 27].
- Type 2 diabetes and hypertension. With increasing obesity class, there is an increasing risk of type 2 diabetes and hypertension, both independent risk factors for COVID-19 severity [23]. The prevalence of type 2 diabetes is markedly increased in various obesity syndromes such as PWS (25–30% in adults with PWS) [51, 52], 16p11.2 deletion syndrome and BBS [53] and ALS [54].
- Respiratory insufficiency. Sleep apnoea is associated with severe obesity. In PWS, risk on sleep apnoea and hypoventilation syndrome from central and obstructive origin is increased [55]. Obstructive sleep apnoea has been associated with severe respiratory complications of COVID-19 [56]. Therefore, patients with monogenic and syndromic obesity, especially PWS patients, could be at an even more increased risk of severe course of COVID-19 if they have obstructive sleep apnoea.
- Impaired immune system is common in patients with LEP and LEPR deficiency. Of the total of 88 LEPR deficient patients that have been reported, 23 (52%) presented with frequent infections, of which 3 died due to infections in childhood. In addition, 2 (5%) had lowered CD4+ T-cell count with compensatory increased B-cell count. One patient (2%) had alterations in immune function [9]. It has to be emphasized, however, that the role that LEP plays in immune signalling is complex. For example, LEP promotes T helper lymphocyte differentiation towards a pro-inflammatory T helper cell 1 phenotype [57]. In addition, LEP-deficient mice have an attenuated inflammatory response and decreased disease severity when contracting inflammatory bowel disease [57]. Therefore, it could even be argued that defective LEP signalling could be protective against the pro-inflammatory phenotype of COVID-19. In contrast LEP deficiency is associated with decreased numbers of lymphocytes and LEP is implicated in various important aspects of acquired immunity, such as stimulating maturation and survival of thymic T cells [58]. The latter might suggest that patients with defective LEP signalling are susceptible to impaired response to- and clearance of SARS-CoV-2. Given the lack of data on this subject the effect of SARS-CoV-2 in these patients remains speculative. For those conditions that are known to be characterized by specific immunological perturbations, we advise physicians to contact the immunologist of the patient for specific advice if these patients contract COVID19.
- Adrenal insufficiency. Various genetic obesity disorders are associated with an increased risk of adrenal insufficiency, such as POMC deficiency [10], PCSK1 deficiency [59] and PWS [60]. All forms of adrenal insufficiency have been associated with increased risk of severe infections needing hospitalization [61] and both the primary and secondary forms have been shown to be associated with increased mortality from infections [62]. If these patients with adrenal insufficiency contract COVID/19, they should be treated in the similar way to that of all other adrenal insufficiency patients. In case that the adrenal function of these patients has not been assessed by the time they develop symptoms and signs suggestive of COVID-19, it is important that they are treated as if they have adrenal insufficiency.
- The risk of infection can be increased by behavioural issues. For instance, in patients with PWS, social distancing and face mask wearing can be difficult given the behavioural and intellectual problems these patients encounter.
- The presence of an intellectual disability can increase the difficulty of care management and care planning. They can have difficulties in expressing concerns or complaints. Especially in patients with PWS it is known that they can have a decreased sense of pain and fever can be absent even when severely ill [63].
- Hypogonadism. Genetic obesity is associated with hypogonadotropic hypogonadism. Both testosterone and oestradiol have proven anti-inflammatory, immunomodulatory and protective effect [64, 65]. It has been shown that low testosterone predicts poor prognosis and mortality in SARS-CoV-2 infected men [65]. Furthermore, preliminary clinical evidence suggests that oestrogens may have a protective role in SARS-CoV-2 infected patients [66]. It is important that patients with genetic obesity and hypogonadotropic hypogonadism are receiving the age and sex-specific replacement therapy.
- While surgical weight-loss treatment might not be recommended in some forms of genetic obesity, some patients with these diseases underwent BS. There is no information on this specific group.
- Malnutrition. Obesity does not protect against malnutrition as has been recently reviewed [67]. Moreover, the overall prevalence of malnutrition is high in COVID-19 patients and hypoalbuminemia is associated with worsening of COVID-19 in hospitalized patients [68]. Therefore, assessment of the nutritional risk and early nutritional management in SARS-CoV-2 infection have been recently emphasized by ESPEN [69]. Quantity of weight loss, reduced muscle mass, reduced food intake and degree of inflammation are major factors that should be assessed and diagnosis of malnutrition and adaptation of nutritional strategy, are important in patients with COVID-19, also in obese patients.

## Further considerations with respect to COVID-19 treatment in patients with genetic obesity

Although the majority of factors important for the treatment of patients with genetic obesity in the context of COVID-19 are addressed in the paragraphs above, there are still some additional considerations to discuss. First of all, the severely increased fat mass in most patients with monogenic and syndromic obesity should be taken into consideration when drugs are prescribed. Specifically, in treatment with fat soluble drugs such as glucocorticoids and certain antibiotics, dosing per metre squared should be considered over normal dosing regimens. In addition, considering the large amount of thromboembolic complications in COVID-19, we stress the importance of taking BMI into account when calculating the dose of prophylactic anticoagulants.

## Corona quarantine measures and genetic obesity

Since the emergence of SARS-CoV-2, many governments have introduced partial or complete lockdown with various measures such as social distancing. Given the increased possibility of a severe course of COVID-19 in people with obesity, the question rises whether extra measures should be taken to prevent people with severe obesity, such as people with genetic obesity, from contracting the disease. On the other hand, the COVID-19 quarantine measures can be a health threat in itself, especially in children. Studies report adverse effects on psychological wellbeing such as anxiety, worrying, irritability, depressive symptoms and post-traumatic stress disorder symptoms in 19–44% of children from the general population [70, 71]. In addition, children with obesity seem to perceive themselves to be vulnerable to COVID-19 because obesity is a risk factor for severe course of the disease. In a Dutch study among children with severe obesity, aged 7–15 years, COVID-19-related anxiety was reported in 32% of children [72]. Self-imposed strict quarantine measures were present in 25% of these families. Given the low incidence of a severe course of COVID-19 in children with obesity and the importance of social interaction and education in general, adherence to the local measures should suffice for children with genetic obesity. Therefore, we do not advice a more strict quarantine regime for this age group at this point in time.

Also, quarantine measures have a considerable impact on lifestyle as observed in an Italian study showing unfavourable changes in eating, sleeping and activity behaviours in children and adolescents with obesity [73]. Many patients report that they experience changes in their home situation, physical activity, sedentary activity, sleep and eating behaviour and these changes could increase the risk of weight gain and comorbidities. Maintaining a healthy lifestyle by engaging in physical activity even indoors and making healthy food choices are strongly encouraged. Ongoing monitoring of obesity is important to maintain during quarantine. In this respect, it is important to note that due to various quarantine measures, a lot of general non-emergency care has come to a standstill. This can have detrimental effects for obesity treatment. It is of utmost importance that during possible future lockdowns, maximum efforts should be undertaken to make sure that obesity treatment and check-ups are continued, using telemedicine options as often as possible. Furthermore, given the importance of physical activity in the treatment of these patients, it is key that regular exercise is promoted within the boundaries of local measures. For example by promoting outdoor physical activity, or increasing the time spent on physical education in schools.

In conclusion, although literature on COVID-19 and genetic obesity is lacking, there is a theoretical basis to assume that these patients have an increased risk for a severe course of COVID-19, especially in case of severe obesity (BMI > 35 kg/m^2^). Maintaining a healthy lifestyle by engaging in physical activity even indoors and making healthy food choices are strongly encouraged. Ongoing monitoring of genetic obesity is important to maintain. During quarantine or possible future lockdowns, maximum efforts should be undertaken to make sure that obesity treatment and check-ups are continued. The most important recommendations are summarized in Box 1.

Box 1. Key principals of the management of patients with genetic obesity during COVID-19 pandemic *Patients that have contracted COVID-19*

Be aware of the possibility of adrenal insufficiency e.g. POMC deficiency, PWS

Be aware of the possibility of a more severe course in patients with concomitant immunodeficiency (e.g. LEP and LEPR deficiency). However defective LEP signalling could also be protective against the pro-inflammatory phenotype of COVID-19

Be aware of disease severity being masked by insufficient awareness of symptoms in syndromic obesity patients with intellectual deficit (in particular PWS)

Adjust medication dose to increased body size, preferably use dosing in m^2^

Be aware of the high risk of malnutrition in patients with Sars-Cov2 infection, even in case of obesity

*Obesity management during the pandemic*

Strive for optimal obesity management and a healthy lifestyle within the possibilities of the regulations to prevent weight (re)gain

Address anxiety within consultations, since prevalence of anxiety for COVID-19 is underestimated

Abbreviations: *POMC* pro-opiomelanocortin, *PWS* Prader-Willi Syndrome, *LEP* leptin, *LEPR* leptin receptor

## Disclaimer

Due to the emerging nature of the COVID-19 crisis, this document is not based on extensive systematic review or meta-analysis, but on literature review and expert consensus. The document should be considered as guidance only; it is not intended to determine an absolute standard of medical care. Healthcare staff need to consider individual circumstances when defining the management plan for a specific patient.
